# A Triple Laser Combination Treatment for Facial Angiofibromata Management in Tuberous Sclerosis and Literature Review

**DOI:** 10.1055/a-2306-0962

**Published:** 2024-06-14

**Authors:** F. Neamonitou, K.K. Neamonitos, S. Stavrianos, K.P. Neamonitos

**Affiliations:** 1 Department of Plastic Surgery, General Anticancer Oncological Hospital of Athens ‘Saint Savvas’, Athens, Greece; 2 Department of Dermatology, General University Hospital of Athens ‘Attikon’, Athens, Greece; 3 Hellenic Society of Dermatology Surgery (HSDS), Private Dermatological Clinic ‘Laserderm Academy’, Athens, Greece

**Keywords:** angiofibroma, Erbium, laser, pulsed dye laser, tuberous sclerosis

## Abstract

Angiofibromas are a common facial manifestation of tuberous sclerosis (TS). However, current treatments have proven ineffective due to high recurrence rates and noncompliance. To address this issue, we developed a new triple laser therapy protocol for more effective management of angiofibromas. We conducted tests to validate its efficacy. This is a prospective study of 10 patients with TS (4 women and 6 men, mean age 26.3 years [15–37 years]) with angiofibromata who received triple sequential laser therapy at our private dermatological clinic conducted from January 2000 to December 2022. We evaluated the outcome with the Facial Angiofibromata Severity Index (FASI) via clinical photography (0, 6 months, 1 year, and 2 years), and Dermatology Life Quality Index (DLQI). All patients had a successful recovery without any complications. Among these 10 patients, 4 experienced localized recurrences at their 6-month follow-up. These recurrences were treated with a second single carbon dioxide laser session. After 2 years of follow-up, we observed no recurring facial cutaneous manifestations. Furthermore, all patients experienced a decrease in their FASI score after treatment. According to the Visual Analogue Scale, patients reported 95% satisfaction, and DLQI indicated only a minor impact on their everyday lives. We believe that this protocol of three-step laser treatment is effective, safe, and compliable for patients with facial angiofibromata, providing a satisfactory outcome adaptable to the daily dermatological and plastic surgery practice.

## Introduction

Tuberous sclerosis (TS) is a rare genetic autosomal dominant neurocutaneous disorder with a prevalence of 1 to 6,000 worldwide, affecting both sexes and all ethnic groups. 1 It is a multisystem syndrome involving the brain, skin, heart, kidneys, eyes, lungs, and liver, which manifests from childhood. 2 The classical triad of TS is seizures, mental retardation, and angiofibromata, but this occurs in only 29% of patients with TS. 3 However, skin involvement is crucial for suspecting the diagnosis of TS. 4 The most common cutaneous lesions in TS are hypomelanotic macule (90%), facial angiofibroma (75%), and Shagreen patch (20–30%), and less manifested periungual or subungual fibroma (Koenen tumor). Linkage studies have shown locus heterogeneity with Tuberous Sclerosis Complex genes (TSC1, TSC2), hamartin, and tuberin mapped to chromosomes 9q34 and 16p13.3 respectively. 5 Facial angiofibromata are often presented by excessive facial flushing in infancy and gradually become more prominent, causing cosmetic and hygienic troubles in adulthood. 6

### Carbon Dioxide Laser

The carbon dioxide laser (CO _2_ laser) is an ablative laser which uses the high absorption of infrared light in water (λ = 10,600 nm), and it is indicated for cutting and vaporizing tissue in a controlled manner. It has many applications in dermatology (condylomata acuminata, common warts, angiofibromata, syringomatas, trichoepitheliomatas, epidermal nevi, acne scars, rhytids, etc.) 7 and it has been used to treat satisfactory angiofibroma, especially when they are exophytic and large. Yet, several studies have demonstrated a high percentage of recurrences. 8 This laser application is accompanied by thermal damage lateral to the area of treatment, which is increased if multiple passes are required.

### Erbium–Yttrium Aluminum Garnet Laser

Erbium–Yttrium Aluminum Garnet (Er–YAG) laser is another ablative laser with λ = 2,940 nm, strongly absorbed by water (absorption coefficient 12,000 vs. 800 cm ^−1^ for CO _2_ ) with less than 50 μm residual damage compared with the 80 to 150 μm typically of pulsed CO _2_ laser exposure. 9 Moreover, its application is wide in skin resurfacing, especially in photodamaged patients with wrinkles, acne scars, and superficial lesions. The Er–YAG laser system can deliver therapeutic intervention in both short and long pulse durations. This versatile technology offers a range of benefits for clinical applications, including precise tissue ablation, reduced thermal damage, and improved postoperative healing. The latter is the most essential advantage as wounds reepithelialize earlier than CO _2_ laser wounds, yet its efficacy is lower. 10

### Pulsed Dye Laser

Pulsed Dye Laser (PDL) technology is a vascular laser of λ ranging from 550 to 610 nm using the principle of selective photothermolysis via light absorption by oxyhemoglobin. 10 11 It is indicated for vascular lesions such as lower extremity spider veins and facial vessels associated with rosacea, photodamage or simply heredity, diffuse erythema (e.g., rosacea), facial telangiectasia, Port Wine Stains, hemangiomas, scars and red striae with less risk of epidermal damage, and hyperpigmentation. 12

## Ideas

### Materials and Methods

This is a prospective study of 10 patients (4 women and 6 men, mean 26.3 years [15–37 years]) with cutaneous TS manifested as angiofibromata on malar, nasolabial regions, dorsum of the nose, and the chin. All patients who were diagnosed with TS, and referred to our clinic from various dermatological outpatient clinics at public hospitals in Greece, received our suggested treatment after providing their informed consent. Only four patients suffered from mild mental retardation, while all experienced at least two episodes of epileptic seizures. They all received a triple laser therapy (CO _2_ laser, Erbium laser—short and long pulse, PDL) in one session at our private dermatological clinic from January 2000 to December 2022. At our clinic, we have treated a total of 14 patients with TS. However, four of them were excluded from the study as they had undergone laser treatments in separate sessions. These four patients were used as a control group to establish our protocol therapy pathway and minimize the number of sessions required. Before treatment, we obtained informed consent from all patients or their guardians in case the patients were unable to give consent. We tested our protocol, which was delivered the same to the remaining 10 patients. The efficacy of the treatment was assessed through a clinical photography-based evaluation of the Facial Angiofibromata Severity Index (FASI) at baseline and 2 years posttreatment. Thus, we employed the information panel of the published article by Salido-Vallejo et al. 13 This comprises seven photographs depicting the complete range of FASI scores from 0 (the best) to 3 (the worst), encompassing erythema (0–3), size (0–3), and extent (0, 2, or 3).

Additionally, we gauged the level of patient satisfaction using the widely accepted Visual Analogue Scale (VAS) scoring system during follow-ups and the Greek version of the Dermatology Life Quality Index (DLQI) before and at 2 years follow-up via telephone interviews (license with ID CUQoL2236 has been granted for the purpose of our study). The DLQI is a tool used to assess the impact of a skin condition on an individual's quality of life. Scores on this scale range from 0 to 30, with a score of 10 or above indicating a significant impairment in daily functioning due to the skin disease. 14

### Operative Procedure-Algorithm

All patients received prescribed topical anesthetic cream preparation CHEMCO® (benzocaine micronized 20%, lidocaine base 6%, tetracaine HCL 4%, propylene glycol, CHEMCO® Lisolate®) for 45 minutes followed by local anesthesia with xylocaine 1% diluted with NaCl 0.9% 1:1, and one ampule of adrenaline 1:100,000. In some cases, we administered a bilateral infraorbital block using 1 to 2 mL of 1% xylocaine on each side. Protective goggles were given to the patient for eye safety.

All 10 patients underwent the three-step methodical laser treatments tailored to each patient according to the lesions' size and depth in one session.

In the first step, topical ablation of the skin lesions was performed with a CO _2_ laser with a pulse duration of 350 μs and frequency of 75 Hz to resurface the diseased skin and flatten the cobblestone area. The second step involved Er–YAG laser short and long pulse in a specific pattern. In more detail, the first pass was performed with the long pulse duration, while the second was with the short. The long pulse enhanced ablation, hemostasis, and skin tightening. In contrast, the short pulse facilitates skin sublimation, and cleanses the surface from skin debris produced by CO _2_ laser and Er–YAG long pulse with minimal thermal damage. Thus, it prepares the skin for vascular laser treatment.

The long pulse settings were pulse duration of 5 or 7 or 10 milliseconds, frequency 10 Hz, and energy 10 to 20 J. The short pulse was 350 μs, 10 Hz frequency, and 10 to 20 J eneergy. The spot size used was 5 to 7 mm, depending on the size of the lesions. The treatment was performed as multiple passes until the pinpoint bleeding was evident ( Table 1 ).

**Table 1:** Three-step algorithm with laser characteristics and settings

Step	Lasers	Wavelength (λ)	settings	Thermal relaxation time comparison	Advantages	Limitation
1	CO _2_	10,600 nm	Pulse duration: 350 μs Frequency: 75 Hz	Thermal relaxation time <1 millisecond	Treating fibrous and protuberant lesions	Small percentage of recurrences if monotherapy, high thermal damage
2 2a 2b	Er–YAG long and short pulse	2,940 nm	Spot size: 5 or 7 mm Pulse duration: 5 or 7 or 10 milliseconds Frequency: 10 Hz Energy: 10–20 J Pulse duration: 350 μs Frequency: 10 Hz energy: 10–20 J	Ablative with thermal relaxation time less than CO _2_ , more collagen tightening Ablative with the least thermal damage, less collagen tightening	Resurfacing with minimal thermal necrosis zone (RTD) Less skin sublimation, collagen tightening, more superficial depth of resurfacing More skin sublimation, cleansing the debris, preparing skin for vascular laser	Not satisfactory for protuberant lesions
3	PDL	585–595 nm	Spot size: 7 mm Energy density: 6–10 J/cm ^2^ (fluency)		1. Treating the vascular component of tuberous sclerosis 2. Preventing early recurrences, erythema, dyspigmentation	No ablation

Abbreviations: CO _2_ , carbon dioxide; Er–YAG, Erbium–Yttrium Aluminum Garnet; PDL, pulsed dye laser; RTD, residual thermal damage.

The last step of the treatment was with PDL with energy density (fluency) 6 to 10 J/cm ^2^ , which was used to collapse the small vessels, and decrease the erythema and dyspigmentation, therefore diminishing the potential recurrence ( Table 1 ).

Posttreatment care included topical fusidic acid ointment, daily local compresses with cool NaCl 0.9% for 7 to 10 days and a silicone-based gel applied onto the skin for 1 to 2 months. Antiherpetic treatment was given only to patients prone to herpetic infection. There is no clear consensus on the requirement of routine prophylactic antibiotherapy before full-field ablative laser applications. However, we prescribed ciprofloxacin as prophylaxis, starting before and continuing 5 days after the treatment. All patients were followed up in 10 days, 1 month, 6 months, 1 year, and 2 years posttreatment.

### Results

**Fig. 1:**
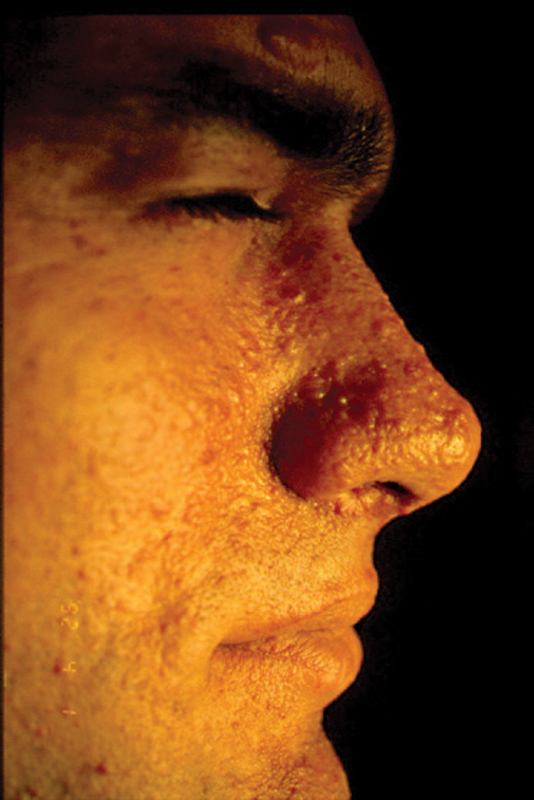
Male, 37-year-old patient with TS preoperative side view. TS, tuberous sclerosis.

All patients completed the study without complications and complied with the treatment and aftercare. None of our patients experienced complications, such as infection, scarring, dyspigmentation, or erythema. According to FASI, our clinical photography database shows a significant improvement in all elements (redness, size, extension) before and after 2 years of treatment. Of 10 patients, 4 presented with few localized recurrences on the 6-month follow-up and a second single CO _2_ laser session was performed. The clinical images of patients were evaluated before and during follow-up visits at 1 and 2 years in our clinic ( Figs. 1 2 3 4 5 ).

**Fig. 2:**
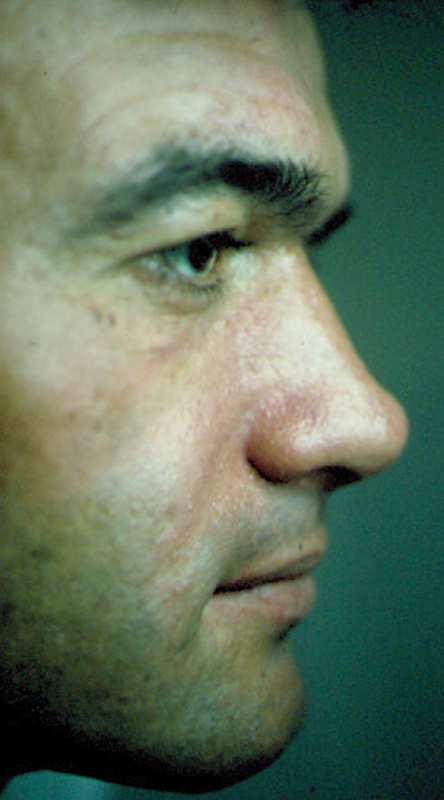
Postoperative side view at 2-year follow-up of the male patient.

**Fig. 3:**
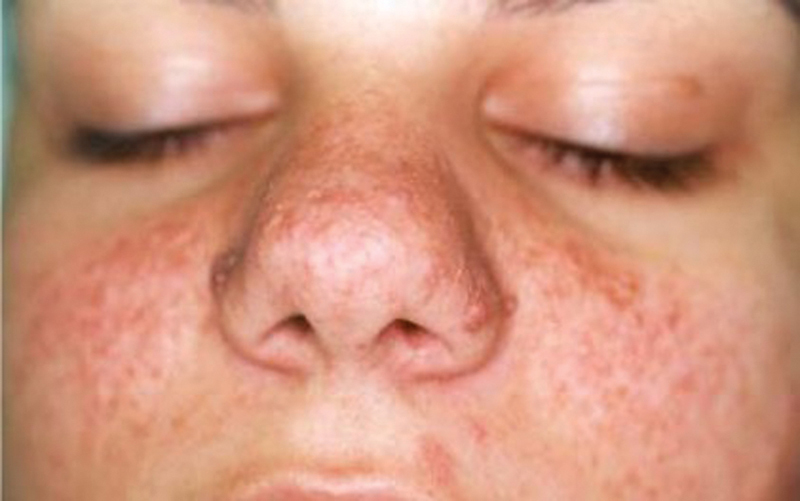
Preoperative enface view of 24-year-old female patient.

**Fig. 4:**
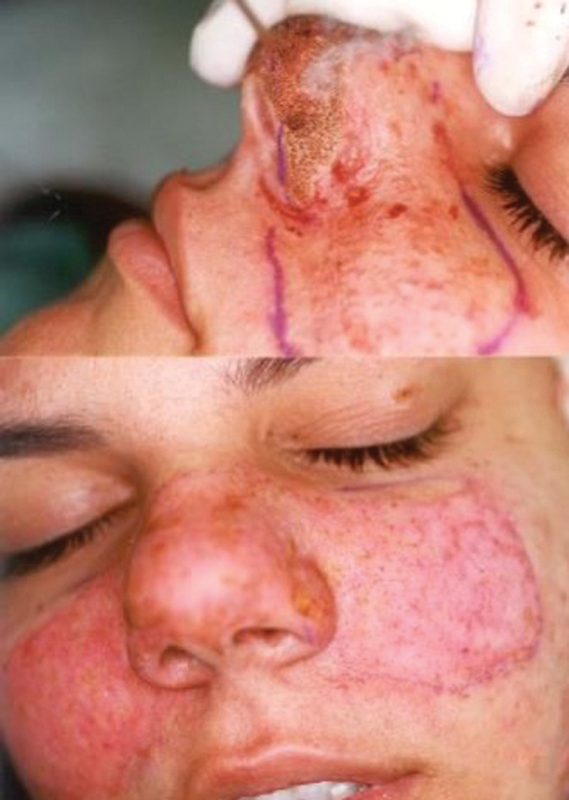
Intraoperative view of the female patient.

**Fig. 5:**
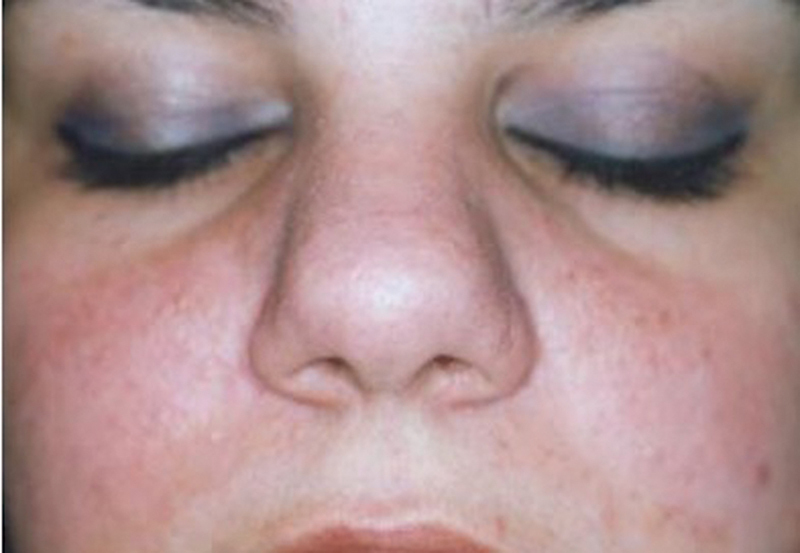
Postoperative enface view at 1-year follow-up of the female patient.

After at least 2 years of follow-up, no patients showed signs of recurring facial angiofibromata. Only four patients had attended the 5-year follow-up; of those, two experienced a mild recurrence of isolated small malar angiofibromata. One of them repeated CO _2_ laser treatment for the few malar angiofibromata, while the other was satisfied with the outcome.

The Fitzpatrick skin type distribution among our patients is as follows: only three patients exhibited skin type 2, one had skin type 4, and the majority presented with skin type 3. Due to the limited population data, no correlation was found between skin type and treatment efficacy ( Table 2 ).

**Table 2:** The table includes information on population demographics, such as age and gender, as well as details on Fitzpatrick type, Facial Angiofibroma Severity Index, and visual analogue scale scores, recurrences, and the length of follow-up periods

Pt No.	Gender	Age	FT	FASI before	FASI 2y	gDLQI before	gDLQI 2y	VAS 2y	Follow-up (y)	Recurrence 6m	Recurrence 2y	Recurrence 5y
1	M	25	3	8	1	10	2	10	2	No	No	N/A
2	M	27	3	8	1	15	3	10	2	No	No	N/A
3	F	15	3	8	1	19	2	10	7	No	No	NO
4	F	22	2	9	2	10	4	7	3	Yes	No	N/A
5	F	31	3	9	1	17	2	10	6	No	No	Yes
6	M	24	2	9	2	11	3	10	2	No	No	N/A
7	F	24	2	8	2	13	4	10	4	Yes	No	N/A
8	M	37	4	8	2	20	2	9	10	Yes	No	Yes
9	M	32	3	9	2	22	5	10	6	Yes	No	NO
10	M	26	3	7	1	11	3	10	2	No	No	N/A

Abbreviations: FASI, Facial Angiofibroma Severity Index; FT, Fitzpatrick type; gDLQI, Greek Dermatology Life Quality Index; m, months; VAS, visual analogue scale; y, years.

According to the VAS survey conducted 2 years posttreatment, 95% of the participants reported high levels of satisfaction with the treatment (rating 7–10/10). The research study involved administering the Greek version of DLQI to individuals before and after a 2-year follow-up period. Prior to receiving treatment, all the patients had been experiencing a significant negative impact on their quality of life due to their skin condition. However, the results of the 2-year follow-up revealed that the condition had a minimal impact on the daily lives of the participants. Detailed findings for each patient can be found in Table 2 .

## Discussion

In light of the literature, there are some case reports and a few case series with angiofibroma management in multiple sessions in TS patients. The first laser application of angiofibromata was reported in 1988 with Argon laser, and the recurrence rates were high on long-term follow-up. 15 Currently, lasers to treat angiofibromata can be mainly classified as ablative and vascular lasers. As ablative lasers, CO _2_ and Er–YAG flatten the exophytic lesions and resurface the affected area, respectively. Both ablative lasers are needed to succeed in the best outcome as CO _2_ manages well the exophytic lesions while the Er–YAG resurfaces the affected area, eliminating the small lesions with minimal residual damage, a trait the CO _2_ laser cannot provide. Regarding vascular lasers, mainly PDL and, to a lesser extent Nd:YAG (neodyniumdopped Yttrium Aluminum Garnet) lasers, are efficient for alleviating excess vascularization and minimizing the potential erythema and hyperpigmentation. Only one case series involves 13 patients treated with a combination of Er–YAG laser, CO _2_ laser, and PDL. However, every laser application treatment was performed in different sessions 3 months apart. 8 To date, no other study provides one-session management of angiofibromata.

There are many other treatment methods in the literature for the management of angiofibromata, such as chemical peels, curettage, electrosurgery, cryotherapy, 16 dermabrasion, 17 and shave excision 18 with a moderate aesthetic outcome, high recurrence rate, and less compliable for the patient. Another modality is topical mTOR inhibitors, which serve as targeted treatment agents for TS regarding the role of the Pi3K–AKT–mTOR (phosphoinositide 3-kinase- protein kinase B-mammalian target rapamycin) pathway in etiopathogenesis. A systematic review tested the efficacy and safety of topical sirolimus, which suggested 0.2% concentration as the most effective and safe in treating facial angiofibromata in patients with TS. However, many patients experienced irritative adverse effects, which led them to withdraw. 19 Furthermore, the efficacy of topical sirolimus indicates that especially large lesions tend to be resistant. Limited data on the combination of topical sirolimus with laser treatments provide a rational alternative for larger lesions, less resistance, less concentration, and are shown to be more effective overall than solely tacrolimus treatment. 20 In our study, 7/10 of our patients had utilized topical sirolimus. Yet, all had to halt this treatment due to persistent irritation, recurrence of issues, and noncompliance. The remaining three patients opted for laser treatment without trying topical therapy despite it being offered. A multimodal approach could potentially provide synergistic benefits for the treatment of angiofibroma. By incorporating adjunct interventions such as topical sirolimus or other targeted agents that modulate the mTOR pathway or integrating multimodal treatments targeting different aspects of angiofibroma pathogenesis, treatment outcomes may be enhanced synergistically. However, it is essential to note that patient compliance poses a challenge as most patients may not be able to tolerate topical therapies and prefer laser therapies that can provide an immediate and long-lasting solution.

It is important to note that our study had certain limitations. First, the rarity of the condition resulted in a relatively small sample size, which may have reduced the power of our analyses and the generalizability of our findings to a broader population. Furthermore, the patients we studied were from diverse regions of Greece, which posed a challenge in conducting a follow-up period of more than 2 years, as only four patients were able to participate. We believe that including cellular and molecular level assessments could have helped in a more comprehensive evaluation of patient outcomes. To include this level of testing in future studies, funding is necessary.

Our goal was to identify a standalone cost-effective treatment pathway for facial angiofibroma with reproducible efficiency. By using two ablative and one vascular lasers during one single session, it is possible to achieve desirable acceptable long-lasting results with no complications. Erbium (short and long pulse) and CO _2_ lasers destructed and resurfaced the area without cicatrization, while PDL managed the vascular component and the likelihood of recurrence. This protocol of three-step laser treatment is effective, safe, and compliant for patients with facial angiofibromata, providing a satisfactory outcome adaptable to the daily dermatological or plastic surgery practice.
